# Mesenchymal stem cell-derived extracellular vesicles affect disease outcomes via transfer of microRNAs

**DOI:** 10.1186/s13287-018-1069-9

**Published:** 2018-11-21

**Authors:** Guanguan Qiu, Guoping Zheng, Menghua Ge, Jiangmei Wang, Ruoqiong Huang, Qiang Shu, Jianguo Xu

**Affiliations:** 1 Shaoxing Second Hospital, 123 Yanan Road, Shaoxing, 312000 Zhejiang China; 2 The Children’s Hospital of Zhejiang University School of Medicine, 3333 Binsheng Road, Hangzhou, 310051 Zhejiang China; 3 The First Affiliated Hospital of Zhejiang University School of Medicine, 79 Qingchun Road, Hangzhou, 310003 Zhejiang China

**Keywords:** Mesenchymal stromal (stem) cells, Extracellular vesicles, microRNA, Exosomes, Microvesicles

## Abstract

Mesenchymal stem cells (MSCs) are adult stromal cells with the capacity to differentiate into multiple types of cells. MSCs represent an attractive option in regenerative medicine due to their multifaceted abilities for tissue repair, immunosuppression, and anti-inflammation. Recent studies demonstrate that MSCs exert their effects via paracrine activity, which is at least partially mediated by extracellular vesicles (EVs). MSC-derived EVs (MSC-EVs) could mimic the function of parental MSCs by transferring their components such as DNA, proteins/peptides, mRNA, microRNA (miRNA), lipids, and organelles to recipient cells. In this review, we aim to summarize the mechanism and role of miRNA transfer in mediating the effects of MSC-EVs in the models of human diseases. The first three sections of the review discuss the sorting of miRNAs into EVs, uptake of EVs by target cells, and functional transfer of miRNAs via EVs. Then, we describe the composition of miRNAs in MSC-EVs. Next, we provide the existing evidence that MSC-EVs affect the outcomes of renal, liver, heart, and brain diseases by transferring their miRNA contents. In conclusion, EV-mediated miRNA transfer plays an important role in disease-modulating capacity of MSCs.

## Background

Mesenchymal stem (stromal) cells (MSCs) are a heterogeneous population of progenitor cells which possess angiogenic, anti-apoptotic, and immunomodulatory properties. Many clinical trials including the work from our group [1] have tested the applications of MSCs in a wide range of diseases [2, 3]. The initial enthusiasm for MSCs in tissue repair was based on the findings that MSCs are able to home to the damaged tissues and differentiate into many types of cells. However, it has been solidly demonstrated that only a small fraction of the transplanted MSCs actually are able to engraft in host tissues. Then, it was proposed that paracrine soluble factors secreted by MSCs were responsible for the mechanism since conditioned medium from MSCs was also able to exert similar effects on the damaged tissues [4]. In recent years, the literature supports that the paracrine mechanism of MSCs is mediated at least in part by extracellular vesicles (EVs) [5]. Also, MSCs are one of the most efficient producers of EVs among different cell types [6].

EVs are membrane-contained vesicles released by most types of cells including MSCs. A crucial criterion of EVs is that they must be harvested from extracellular fluids such as tissue culture or bodily compartment through ultracentrifugation based on the position statement from the International Society for Extracellular Vesicles (ISEV) [7]. EVs can be broadly classified into three subtypes based upon vesicle sizes and methods of cellular release: exosomes (30–130 nm), microvesicles (100–1000 nm), and apoptotic bodies (50–4000 nm). Exosomes are produced after the fusion of multivesicular bodies (MVB), which are endocytic organelles containing many luminal vesicles, with the plasma membrane. On the other hand, both microvesicles and apoptotic bodies are formed by direct budding from the plasma membrane. There is a lack of distinctive approaches to separate the three subtypes of EVs as they have overlapping size, density, and membrane composition [8]. EVs have been tested as disease biomarkers since EVs reflect the status of their parent cells [9]. Furthermore, EVs have the capacity to transfer molecules, such as DNA, proteins, mRNA, microRNA (miRNA), lipids, and organelles, from their originating cells to the recipient cells and serve as a mechanism for intercellular communication. More and more evidences reveal that EVs mediate the effects of parental cells via miRNA delivery [10].

The majority of total RNA content in EVs includes tRNAs, mRNAs, miRNAs, and long non-coding RNAs. Sequence analysis showed that approximately 13% of the total RNA content of EVs from milk is miRNAs [11]. miRNAs are 19–24 nucleotide non-coding RNAs, which modulate the expression of up to 30% of all mammalian protein-encoding genes [12]. In the biogenesis of miRNAs, primary miRNAs (Pri-miRNAs), containing cap structures and poly(A) tails, are transcribed by RNA polymerase II. Pri-miRNAs are cleaved by the RNAse Drosha into hairpin precursor miRNAs with 60–70 nucleotides (pre-miRNAs) in the nucleus. Pre-miRNAs are then cleaved into double-strand mature miRNAs by another RNase Dicer in the cytosol [13]. Next, one strand of mature miRNAs associates with agonaute 2 (ago2) and forms the RNA-induced silencing complex (RISC). The miRNA in the complex is then able to interact primarily with 3′-untranslated region of target mRNAs via base pairing complementarity and leads to target degradation or translational repression [14]. This article reviews the existing literature on the sorting and trafficking of miRNAs as well as the role of miRNA transfer in mediating the effects of MSC-derived EVs (MSC-EVs) in diseases of vital organs, including the kidney, heart, liver, and brain.

## Sorting of miRNAs into EVs

Many studies have shown that miRNAs are selectively sorted in EVs. Such sorting of miRNAs was demonstrated in PC-3 prostate cancer cells in which miRNAs with a low number in their names were found to be underrepresented in EVs [15]. In dendritic cells, sequencing analysis showed that some miRNAs in EVs were enriched when compared with the cellular miRNAs [16]. Selective enrichment of miRNAs including Th2 inhibitory miRNAs into EVs was revealed in bronchoalveolar lavage of mice after horse dust mite exposure. Increased release of EVs into the airway was involved in the pathogenesis of dust mite allergen-induced airway inflammation [17]. Furthermore, when THP-1 cells were transfected with chemically modified miR-143, abundant modified miR-143 was identified in EVs of the culture medium [18].

The secretory mechanism of miRNAs has been postulated and is regulated by a ceramide-dependent pathway. Ceramide is synthesized by neutral sphingomyelinase 2 (nSMase2) and controls the secretion of EVs. Decreased synthesis of ceramide via nSMase2 inhibition led to reduced secretion of miRNAs. Overexpression of nSMase2 elevated the extracellular miRNA levels [19]. However, the mechanisms behind the miRNA sorting are not well understood. Mechanistically, the loading of miRNAs into EVs may rely on factors including the binding motifs of the miRNAs and the miRNA-associated proteins such as Argonaute 2 (Ago2), Alix, and MEX3C (Table 1). For the miRNA motifs, the heterogeneous nuclear ribonucleoprotein A2B1 (hnRNPA2B1) controlled the packaging of miRNAs into EVs via binding the recognition motif GGAG of miRNAs. Mutagenesis of the binding motifs or alteration of hnRNPA2B1 sumoylation altered the loading [20]. Another motif-binding protein, the synaptotagmin-binding cytoplasmic RNA-interacting protein (SYNCRIP), recognized GGCU motif in miRNAs. Knockdown of SYNCRIP impaired the sorting of miRNAs into EVs. Adding the GGCU motif into a poorly exported miRNA promoted its sorting into EVs [21]. Another study showed that 3′-uridylated miRNAs were enriched in EVs as compared with parental B cells while the mechanism is unknown [22]. In terms of the miRNA-associated proteins, Ago2 is an effector protein known to bind with miRNAs to form the RISC. Then, RISC is able to associate with MVB which produce EVs after fusion with the plasma membrane, indicating that Ago2 could control the sorting of miRNAs [23]. Both Ago2 level and Ago2 phosphorylation by MEK-ERK regulated the sorting of *let-7a*, *miR-100*, and *miR-320a* into the EVs [24]. Alix, which is known to associate with the endosomal sorting complex required for transport (ESCRT) and the generation of EVs, has been reported in participating in miRNA packing. Knockdown of Alix did not affect the number of EVs released by human liver stem-like cells, but it significantly reduced the miRNA levels in the EVs [25]. However, knockdown of ESCRT proteins did not affect miRNA sorting to EVs, indicating ESCRT is not involved in miRNA packing [19]. Finally, MEX3C is a RNA-binding ubiquitin E3 ligase and interacts with adaptor-related protein complex 2 (AP-2), a cargo adaptor in clathrin-mediated endocytosis. Inhibition of the MEX3C or AP-2 decreased the level of miR-451a in EVs but not cellular miR-451a [26].

**Table 1:** Mechanisms for miRNA sorting

References	Regulating factor	Mechanisms	miRNA examples
Villarroya-Beltri et al. 2013 [20]	GGAG motif	Nuclear ribonucleoprotein A2B1 binds GGAG in miRNAs.	miR-198 and miR-601
Santangelo et al. 2016 [21]	GGCU motif	Synaptotagmin-binding cytoplasmic RNA-interacting protein (SYNCRIP) recognizes GGCU motif in miRNAs.	miR-3470a and miR-194-2-3p
Koppers-Lalic et al. 2014 [22]	3′-End uridylation	3′-End posttranscriptional modification with uridylation demarcates EV miRNAs.	miR-486-5p
Gibbings et al. 2009 [23] and McKenzie et al. 2016 [24]	Ago 2	1. Ago2 binds with miRNAs to form the RISC. 2. RISC associates with MVB to control miRNA sorting.	let-7a, miR-100, and miR-320a
Iavello et al. 2016 [25]	Alix	Alix binds to Ago2 and miRNAs.	miR-24, miR-31, and miR-125b
Lu et al. 2017 [26]	MEX3C	MEX3C binds with AP-2 and promotes miR-451a sorting.	miR-451a

*miRNAs* microRNAs, *EV* extracellular vesicle, *Ago2* argonaute 2, *RISC* RNA-induced silencing complex, *MVB* multivesicular bodies, *AP-2* adaptor-related protein complex 2

## Uptake of EVs by target cells

The contents of EVs may gain entry into target cells via two mechanisms: endocytosis and fusion. The primary entry mechanism of EVs is endocytosis, including clathrin-dependent endocytosis and clathrin-independent pathways which are mediated by caveolin, macropinocytosis, phagocytosis, and lipid raft [27]. A population of EVs may be internalized by more than one mechanism depending on the cell types and composition of EVs [28]. The selection of target cells is mediated by surface receptors and adhesion molecules (i.e., tetraspanins, integrins, proteoglycans, and lectins) on both EVs and target cells. Rana et al. reported that EVs expressing tetraspanin 8 were preferentially taken up by endothelial and pancreatic cells [29]. The second entry mechanism of EVs is a direct fusion of the EV membrane with the cell plasma membrane [30]. Parolini et al. showed that the fusion of labeled EVs with melanoma cells was enhanced under acidic conditions [30]. Montecalvo et al. demonstrated that spontaneous transfer of EVs occurred between dendritic cells via fusion and release of the content into the cytosol [31].

Mechanisms for uptake of MSC-EVs by target cells have also been investigated in several studies. In a model of myocardial infarction, Arslan et al. reported that MSC-EVs were internalized by cardiomyocytes at the infarct site via endocytosis or phagocytosis. They also demonstrated that homogenized EVs failed to reduce the infarct size [32]. Vonk et al. found that MSC-EVs were taken up by chondrocytes from osteoarthritic patients as short as 30 min of incubation. In addition, MSC-EVs co-localized with late endosomal marker LAMP-1 [33]. Stik et al. documented that MSC-EVs were selectively taken up by hematopoietic stem cells ex vivo and in vivo and maintained the survival and clonogenic potential of hematopoietic stem cells by preventing apoptosis [34].

## Functional transfer of miRNAs via EVs

Many reports have documented that miRNAs packaged in EVs can affect the function of the recipient cells. Valadi et al. were the first to report that EVs contained transferable and functional mRNAs and miRNAs, showing that new mouse proteins were discovered in the recipient cells after transferring the content of mouse EVs to human mast cells [35]. Pegtel et al. showed that Epstein-Barr viral miRNAs were secreted from infected B cells via EVs and transferred to dendritic cells, resulting in miRNA-mediated repression of EBV target genes such as *CXCL11/ITAC* [36]. In another study of functional transfer of miRNAs, nematode miRNAs were discovered in mouse cells when incubated with nematode EVs, leading to the suppression of target gene Dusp1. In vivo, type 2 innate responses were suppressed when nematode EVs were administered to mice [37]. In addition, Zhou et al. revealed that when plasma EVs from erythropoietin-treated mice were cultured with fibroblasts, miR-144 levels in fibroblasts were elevated in a dose-dependent manner, resulting in reduced expression of tissue plasminogen activator. However, the expression of pre-miR-144 was unchanged, indicating that the miR-144 increase is the result of transfer from EVs to fibroblasts rather than de novo synthesis [38]. Transfer of functional miRNAs is also manifested in MSC-EVs. miR-146a was upregulated by treating MSCs with IL-1β and selectively packaged into MSC-EVs. Coculture of the MSC-EVs with macrophages led to an elevated level of miR-146a and M2 polarization, which was blocked by miR-146a inhibitor [39].

## Composition of miRNAs in MSC-EVs

Several studies have compared the contents of miRNAs between MSC-EVs and their parental MSCs. Shao et al. reported that the expression of miR-21 and miR-15, which inhibits cardiac functions, was significantly lower in MSC-EVs compared to that of MSCs [40]. miRNA array analysis revealed that miRNA profiles for human glioma-associated MSCs-EVs and human bone marrow MSC-EVs were significantly different from their parental cells. In addition, a group of 8 miRNAs was enriched in glioma-associated MSCs-EVs compared to the parental cells [41]. Baglio et al. showed that the top 4 miRNAs enriched in MSC-EVs compared to MSCs were miR-4485, miR-150-5p, miR-6087, and miR-486-5p. On the other hand, the top 4 miRNAs overrepresented in the MSCs were miR-34a-5p, miR-34c-5p, miR-15a-5p, and miR-136-3p [42]. In MSC-EVs derived from porcine adipose tissue, miRNA sequence analysis annotated a total of 413 miRNAs. However, only miR-183, miR-378, miR-140-3p, and miR-222 were more abundant in EVs compared to MSCs. Further analysis showed that this set of miRNAs targets transcription factors such as SMAD family member 2, POU class-2 homeobox 1, and One cut homeobox 2. The results indicate that miRNAs in MSC-EVs from the porcine adipose tissue may function via modulating transcription factors of recipient cells [43].

There is a lack of a consensus miRNA signature among MSC-EVs from different sources. Table 2 listed miRNAs that are the most highly expressed in the MSC-EVs examined. Baglio et al. compared the miRNA composition of MSC-EVs from adipose- and bone marrow-derived MSCs. The top 5 most abundant miRNAs in the adipose-derived MSC-EVs were miR-486-5p, miR-10a-5p, miR-10b-5p, miR-191-5p, and miR-222-3p. On the other hand, miR-143-3p, miR-10b-5p, miR-486-5p, miR-22-3p, and miR-21-5p were among the most abundant for bone marrow-derived MSC-EVs [42]. In a study from MSC-EVs of human umbilical cord-derived MSCs using high-throughput RNA sequencing, top 8 abundant miRNAs accounted for 40.7% of the total miRNAs. Functional analysis demonstrated that 4 of the 8 miRNAs (miR-21, miR-23a, miR-125b, and miR-145) were essential in suppressing myofibroblast formation by blocking the transforming growth factor-β2/SMAD2 pathway in wound healing [44]. In another study, miRNA profiling analysis of human bone marrow MSC-EVs revealed 171 miRNAs. The top 23 miRNAs made up 79.1% of all the miRNAs, while the rest 148 miRNAs represented a very small percentage of the total reads (0.03 to 0.7%). The top 23 miRNAs target 5481 genes with high stringency by prediction. At system level, the targeted genes participate in angiogenesis. The targeted pathways include Wnt signaling, pro-fibrotic signaling, cell proliferation, and apoptosis [45]. Luther et al. reported that miR-21a-5p was the most highly expressed miRNAs from EVs of mouse bone marrow-derived MSCs and mediated the cardioprotection by MSCs [46]. Lack of a consensus miRNA profile among MSC-EVs may indicate that the expression of miRNAs is influenced by many factors and that individual miRNA may work synergistically to mediate the effects of MSC-EVs.

**Table 2:** miRNAs expression profile of MSC-EVs from different studies

References	Sources of MSCs	Most highly expressed miRNAs	Comments
Baglio et al. 2015 [42]	Human adipose-derived MSCs, passages 2–3	miR-486-5p, miR-10a-5p, miR-10b-5p, miR-191-5p, and miR-222-3p	The five most abundant miRNAs accounted for 43–59% of the total miRNA reads.
Baglio et al. 2015 [42]	Human BM-derived MSCs, passages 2–3	miR-143-3p, miR-10b-5p, miR-486-5p, miR-22-3p, and miR-21-5p	
Fang et al. 2016 [44]	Human umbilical cord-derived MSCs, passages 2–5	miR-21-5p, miR-125b-5p, miR-23a-3p, miR-100-5p, let-7f-5p, let-7a-5p, miR-145-5p, and miR-1260b	The top 8 miRNAs account for 40.7% of total miRNAs.
Ferguson et al. 2018 [45]	Human BM-derived MSCs, passages 1–7	miR-1246, miR-23a-3p, miR-451a, miR-125b-5p, miR-199a-3p, let-7a-5p, miR-4454, and miR-21-5p	The top 23 miRNAs account for 79.1% of total miRNAs.
Luther et al. 2018 [46]	B6 mouse BM-MSCs	miR-21a-5p, miR-486b-5p, miR-486a-3p, miR-143-3p, miR-92a-3p, miR-486a-5p, miR-486b-3p, and miR-22-3p	

*MSCs* mesenchymal stem cells, *EVs* extracellular vesicles, *BM* bone marrow

## MSC-EV-mediated transfer of miRNAs in renal diseases

Cantaluppi et al. were the first to show that EVs from endothelial progenitor cells alleviated kidney injury from ischemia-reperfusion via transfer of miRNAs to resident renal cells [10]. Since then, many groups have examined whether the same mechanism also applies to MSC-EVs in renal and other diseases in vivo (Table 3) and in vitro. EVs from MSCs with global downregulation of miRNAs via Drosha knockdown had lower miRNA content [47]. In a mouse model of glycerol-induced acute kidney injury, intravenous administration of MSC-EVs resulted in morphologic and functional recovery, while the Drosha knockdown counterparts were not effective [48]. In an in vitro model of ischemia-reperfusion injury induced by ATP depletion, Lindoso et al. examined the mechanism of MSC-EVs on miRNA upregulation in renal proximal tubular epithelial cells. Both EV-mediated miRNA transfer and transcriptional regulation of miRNAs in tubular epithelial cells were involved in the process. The predicted targets of involved miRNAs included genes associated with apoptosis, reorganization of the cytoskeleton, and hypoxia [49]. Wang et al. reported that MSCs with miRNA-let7c overexpression homed to damaged kidneys and elevated miR-let7c levels in the kidney in a mouse model of unilateral ureteral obstruction. In vitro, miR-let7c was transferred from MSCs to kidney tubular epithelial cells via EVs. The upregulated miR-let7c targeted TGF-β receptor 1 and attenuated renal fibrosis [50]. In a rat model of acute renal ischemia-reperfusion injury, treatment of MSC-EVs inhibited mitochondria fission in tubular epithelial cells, restored miR-30 levels in injured kidney, and reduced apoptosis. The protective effect of MSC-EVs was lost when treated with miR-30 antagomirs [51].

**Table 3:** Studies demonstrating the MSC-EV-mediated transfer of miRNAs in animal models

References	Disease model	Treatment with MSC-EVs or MSCs, dose, duration, and route	Major findings	miRNA transferred	Target proteins
Collino et al. 2015 [48]	Mouse, rhabdomyolysis-induced acute kidney injury	MSC-EVs with Drosha knockdown or control, 2.2 × 10^8^ EV particles, on day 3 of injury, tail veil	Drosha knockdown blocked morphologic and functional recovery in acute kidney injury	Reduction in all miRNAs in EVs from Drosha knockdown	ND
Wang et al. 2016 [50]	Mouse, renal fibrosis from ureteral obstruction	MSCs with miR-let7c overexpression, 1 × 10^6^ cells, 0 h after obstruction, intravenous	Attenuated kidney injury and fibrosis	miR-let7c	TGF-β receptor 1
Gu et al. 2016 [51]	Rat, renal ischemia-reperfusion injury	MSC-EVs, 100 μg, 0 h after reperfusion, intravenous	Protect the kidney from the injury by inhibition of mitochondrial fission	miR-30	DRP1
Feng et al. 2014 [52]	Mouse, MI	MSC-EVs from ischemic precondition, 1 μg, 0 h after ischemia, intramyocardium	Reduced infarct size	miR-22	Methyl-CpG-binding protein 2
Yu et al. 2015 [53]	Rat, MI	EVs from MSCs with GATA-4 overexpression, harvested from 4 × 10^6^ MSCs, 0 h after ischemia, intramyocardium	Reduced infarct size, promoted cardiac function recovery	miR-19a	PTEN
Wang et al. 2015 [54]	Mouse, sepsis-induced cardiac dysfunction	MSC-EVs, 2 μg/g body weight, 1 h after cecal ligation and puncture model, intravenous	Protection against cardiac dysfunction, apoptosis, and inflammatory response	miR-223	Sema3A and Stat3
Lou et al. 2015 [56]	Nude mouse, hepatocellular carcinoma	MSC-EVs, 50 μg, intra-tumor injection	Increased the antitumor efficacy of sorafenib on hepatocellular carcinoma	miR-122	Cyclin G1 and IGF1R
Lou et al. 2017 [57]	Mouse, CCl4-induced liver fibrosis	MSCs with miR-122 overexpression, 1 × 10^5^ cells, 1 day after the fourth injection of CCl4, tail vein	Alleviated liver fibrosis and collagen deposition	miR-122	Cyclin G1, IGF1R, and P4HA1
Chen et al. 2018 [58]	Mouse, experimental autoimmune hepatitis	EVs from MSCs with miR-223 overexpression; dose not shown; on days 21, 28, and 35 of 42-day experiment; intraperitoneally	Reduced inflammation and reversed liver injury	miR-223	NLRP3
Qu et al. 2017 [59]	Mouse, CCl4-induced liver fibrosis	EVs from MSCs with miR-181 overexpression, 40 μg, twice weekly for 8 weeks, intrasplenic injection	Inhibited liver fibrosis and activated autophagy	miR-181	Bcl-2 and Stat3
Xin et al. 2012 [60]	Rat, stroke from middle cerebral artery occlusion	MSCs, 3 × 10^6^ cells, 24 h postischemia, tail vein	MSCs increased miR-133b level in the brain, MSC-EVs elevated neurite outgrowth in vitro	miR-133	RhoA (candidate)
Xin et al. 2013 [61]	Rat, stroke from middle cerebral artery occlusion	MSCs with miR-133 overexpression or knockdown, 3 × 10^6^ cells, 24 h postischemia, tail vein	miR-133 enhanced axonal plasticity and neurite remodeling	miR-133	Connective tissue growth factor and RhoA
Xin et al. 2017 [62]	Rat, stroke from middle cerebral artery occlusion	EVs from MSCs with miR-17-92 cluster expression, 100 μg, 24 h postischemia, intravenous	Increased neural plasticity and functional recovery	miR-17-92 cluster	PTEN

*MSCs* mesenchymal stem cells, *EVs* extracellular vesicles, *MI* myocardial infarction, *IGF1R* insulin-like growth factor receptor 1, *P4HA1* prolyl 4-hydroxylase alpha 1, *ND* not determined

## MSC-EV-mediated transfer of miRNAs in cardiac diseases

In a mouse model of myocardial infarction, MSC-EVs alleviated cardiac injury. In vitro, EVs enriched with miR-22 were produced by MSCs after ischemic precondition, transferred to cardiomyocytes, and reduced ischemia-induced cardiomyocyte apoptosis. The protective effect of miR-22 was facilitated by target protein methyl-CpG-binding protein 2 [52]. In a rat model of myocardial infarction, EVs from MSCs with GATA-4 overexpression reduced infarct size when injected to the border of an ischemic region. The MSC-EVs were enriched with miR-19a and internalized by cardiomyocytes in vitro. miR-19a inhibitor blocked the protective effects of MSC-EVs. Furthermore, miR-19a targeted PTEN, resulting in the activation of the Akt and ERK [53]. In a mouse model of polymicrobial sepsis, MSC-EVs with miR-223 knockout exacerbated cardiac dysfunction, while wild-type had protective effects. It was proposed that wild-type MSC-EVs delivered miR-223 to cardiomyocytes which downregulated Sema3A and Stat3, two genes regulating inflammation and cell death [54]. Recently, another group reported that MSCs treated with hydrogen peroxide produced EVs with higher miR-21 than control. C-kit+ cardiac stem cells treated with the MSC-EVs had a higher level of miR-21, lower expression of PTEN (miR-21 target protein), and lower oxidative stress-induced apoptosis as compared with the control. Furthermore, the effect of MSC-EVs was blocked by a miR-21 inhibitor [55].

## MSC-EV-mediated transfer of miRNAs in liver diseases

Similar findings have also been documented in liver diseases. In a study of hematoma cells, Lou et al. reported that miR-122 was effectively packaged into EVs of MSCs with miR-122 overexpression, transferred into hepatoma cells, and reduced the expression of target genes, such as cyclin G1 and insulin-like growth factor receptor 1 in the cancer cells. In vivo, the MSC-EVs sensitized hepatoma cells to chemotherapeutic agent sorafenib [56]. The same group also documented that miR-122 was transferred from the MSC-EVs with miR-122 overexpression to hepatic stellate cells and inhibited the expression of its target genes. These genes included insulin-like growth factor receptor 1, Cyclin G1, and prolyl-4-hydroxylase α1, which promoted the synthesis of collagen in hepatic stellate cells. In vivo, MSCs with miR-122 overexpression enhanced the therapeutic effect of MSCs in mitigating carbon tetrachloride-induced liver fibrosis [57]. In a mouse model of experimental autoimmune hepatitis, Chen et al. found that MSC-EVs and MSC-EVs with lentiviral transduction of miR-223 alleviated liver injury, while EVs from MSCs treated with miR-223 inhibitor lost the protective effect. EVs from MSCs transduced with miR-223 reduced the levels of target protein NLRP3 and caspase-1 in vitro [58]. In a mouse model of CCl4-induced liver fibrosis, EVs from MSCs with miRNA-181-5p overexpression alleviated liver fibrosis via activation of autophagy. In vitro, transfer of miRNA-181-5p from MSCs to mouse hepatic stellate cells was shown to be mediated by EV uptake [59].

## MSC-EV-mediated transfer of miRNAs in brain diseases

Xin et al. reported that MSC treatment of rats with stroke elevated miR-133b level in the ipsilateral hemisphere. In vitro, miR-133b was transferred from MSC-EVs to primary cultured neurons and astrocytes. Cultured neurons treated with EVs from MSCs exposed to brain extracts of middle cerebral artery occlusion significantly induced the length branch number and length [60]. The group also documented that infusion of MSCs with miR-133b overexpression, compared with wild-type MSCs, enhanced functional recovery and promoted neurite remodeling in ischemic boundary zone of rats with stroke. In addition, the expression of RhoA, a target protein for miR-133b, was significantly diminished in the ischemic boundary zone after treatment of MSCs with miR-133b overexpression. Furthermore, when rats were treated with MSCs expressing GFP fusion protein containing CD63, a marker for EVs, EVs were released from MSCs and transferred to neighboring astrocytes and neurons [61]. Recently, the same group showed that treatment of stroke rats with tailored EVs containing miR-17–92 cluster enrichment enhanced functional recovery of the brain via downregulation of PTEN expression [62].

miRNA transfer also mediates the effects of MSC-EVs in brain tumors. Figueroa et al. found that EVs from glioma-associated MSCs elevated proliferation and tumorigenicity of glioma stem-like cells in vivo. miRNA profile analysis identified that miR-1587 mediated the effects of MSC-EVs on glioma stem-like cells, in part via downregulation of the nuclear hormone receptor corepressor-1 [41]. In another study, when glioblastoma cells were cultured in the presence of MSCs with anti-miR-9 overexpression, resistance to temozolomide was reversed as evidenced by increased cell death and caspase activity. Flow cytometry study revealed that Cy5 fluorescence could be detected in glioblastoma cells when incubated with EVs from MSCs transfected with anti-miR-9-Cy5, indicating a direct transfer of anti-miR-9 from EVs to glioblastoma cells [63]. Ono et al. reported that breast cancer BM2 cells acquired dormant phenotypes with suppressed proliferation and invasion after culturing with MSC-EVs. RT-PCR analysis revealed that miR-23b was more abundant in EVs from MSCs as compared with fibroblasts. Overexpression of miR-23b in BM2 cells led to dormant phenotypes via inhibiting MARCKS, resulting in reduced cell cycling and motility [64].

## Conclusions

Over the past decade, MSC-EV-based therapies have shown promising results in animal models for a myriad of diseases. MSC-EVs exert their effects mainly via immunomodulation, tissue regeneration, anti-apoptosis, and regulation of tumor progression. MSC-EVs are cell-free products and reduce the risks associated with the use of native or engineered MSCs. The effects of MSC-EVs are of equal potency to those observed with whole MSCs. Therefore, MSC-EVs may be a promising alternative to MSC therapy. As with other cell types, the main functional RNA components in MSC-EVs are miRNAs, which are selectively sorted into EVs and protected from degradation. The release of MSC-EVs allows cells to communicate with other adjacent or distant cells by transferring miRNAs and other biologically active molecules into recipient cells via endocytosis and/or fusion. The effects of MSC-EVs are dependent on the profiles of their miRNAs, which regulate the expression of multiple target genes and participate in various cell signaling processes. However, there is a wide knowledge gap between miRNAs in MSC-EVs and altered gene expression in the target cells. Foremost, it remains unknown what the miRNA signatures of MSC-EVs are. There has not been any individual miRNA or miRNA panel that has been reported as a consensus miRNA profile of MSC-EVs. The lack of miRNA signatures may result from small sample sizes and heterogeneity in methodology, culture condition, cell origin, and cell status. It remains unclear how diverse miRNA profiles in different MSC-EVs exert similar protective function in animal models. Secondly, the sorting mechanisms of miRNAs into MSC-EVs have not been determined. The existing sorting pathways proposed in the literature apply to only a limited number of miRNAs and to some cell types. A general mechanism of miRNA sorting into EVs or MSC-EVs is still unavailable. Lastly, the principle target genes of miRNAs in MSC-EVs also remain unspecified. Based on the bioinformatics tools, dozens or hundreds of genes are potential targets of a single miRNA. Many of the target genes could work independently, synergistically, or even antagonistically. It would be difficult to attribute an observed miRNA-dependent phenotype to a single target as reported in most literature. Further studies may pave the way to develop MSC-EVs therapeutics based on miRNA delivery.

## Abbreviations

Ago 2: Agonaute 2
ESCRT: Endosomal sorting complex required for transport
EVs: Extracellular vesicles
hnRNPA2B1: Heterogeneous nuclear ribonucleoprotein A2B1
ISEV: International Society for Extracellular Vesicles
miRNA: microRNA
MSC-EVs: MSC-derived EVs
MSCs: Mesenchymal stromal cells
MVB: Multivesicular bodies
nSMase2: Neutral sphingomyelinase 2
Pri-miRNAs: Primary miRNAs
RISC: RNA-induced silencing complex
SYNCRIP: Synaptotagmin-binding cytoplasmic RNA-interacting protein
